# A retrospective review of sagittal split ramus osteotomy: Incidence and risk factors for neurosensory disturbance of the inferior alveolar nerve

**DOI:** 10.1371/journal.pone.0348209

**Published:** 2026-05-04

**Authors:** Takuma Watanabe, Shu Inoue, Risa Okada, Ryuji Uozumi, Michinobu Sasaki, Tatsuya Kawamura, Shizuko Fukuhara, Makoto Hirota

**Affiliations:** 1 Department of Oral and Maxillofacial Surgery, Graduate School of Medicine, Kyoto University, Kyoto, Japan; 2 Department of Industrial Engineering and Economics, Institute of Science Tokyo, Tokyo, Japan; 3 Department of Oral and Maxillofacial Surgery, Kyoto City Hospital, Kyoto, Japan; Università degli Studi del Piemonte Orientale Amedeo Avogadro: Universita degli Studi del Piemonte Orientale Amedeo Avogadro, ITALY

## Abstract

Sagittal split ramus osteotomy (SSRO) is the most commonly performed mandibular procedure in orthognathic surgery. Although generally safe, neurosensory disturbance (NSD) of the inferior alveolar nerve (IAN) remains a frequent complication that can impair oral function and quality of life. This retrospective study included 463 patients who underwent SSRO, comprising 873 rami, at our department between 2014 and 2024. Patient- and ramus-level variables were extracted from electronic medical records, with postoperative NSD of the IAN defined as the primary outcome. We investigated the presence or absence of mandibular canal (MC)–lateral cortical bone (LCB) contact on preoperative computed tomography, as well as the three types of lateral osteotomy line on postoperative panoramic radiographs. Descriptive statistics were used to summarize clinical characteristics, and the incidence of NSD was evaluated. Trends in annual NSD proportions were analyzed using a Cochran–Armitage trend test, and univariable and multivariable logistic regression analyses were performed to assess associated factors. The incidence of NSD was 5.6% at the patient-level and 4.0% at the ramus-level. A decreasing trend in annual NSD incidence was observed (p < 0.001). In multivariable analysis, the presence of MC–LCB contact was strongly associated with NSD (odds ratio, 4.96; 95% confidence interval, 2.41–10.06; p < 0.001). The incidence of NSD after SSRO was approximately 5% and showed a declining annual trend over time, possibly reflecting increasing surgical experience and improved instrumentation. MC–LCB contact was associated with an approximately fivefold higher risk of NSD, highlighting the importance of careful and gentle osteotomy.

## Introduction

Sagittal split ramus osteotomy (SSRO), first described in the English literature by Trauner and Obwegeser in 1957, is the most widely used mandibular surgical technique in orthognathic surgery [1,2]. The majority of patients undergoing SSRO—a safe and versatile procedure—are young and have high expectations regarding functional and aesthetic outcomes; however, SSRO is associated with several common complications [3].

Among these, neurosensory disturbance (NSD) of the inferior alveolar nerve (IAN) remains one of the most frequently reported complications [4–8]. Altered sensation of the lower lip and chin may interfere with normal oral functions, including eating, drinking, and speaking; consequently, NSD of the IAN can adversely affect patients’ quality of life both physically and psychologically [9].

NSD of the IAN after SSRO is commonly attributed to mechanical injury to sensory nerve fibers occurring during medial soft-tissue dissection of the mandibular ramus, osteotomy, splitting, or fixation procedures [4]. NSD after SSRO is considered multifactorial, with reported risk factors including age, sex, nerve exposure and manipulation, the magnitude of mandibular movement, mandibular anatomy, surgeon-related factors, cutting and splitting instruments, and the method and timing of postoperative neurosensory assessment [4–7,9–12]. A study reported that 74% of patients undergoing SSRO experienced neurosensory discomfort postoperatively based on subjective assessments [13]. Owing to variability in contributing factors and heterogeneity among study populations, the reported incidence of NSD following SSRO has varied widely, ranging from 9.0% to 84.6%, and remains insufficiently evaluated [3,5].

In this study, we retrospectively reviewed SSRO cases treated at our department over an 11-year period and investigated patient- and ramus-level variables. We analyzed the incidence and annual proportional trends of NSD, as well as the associations between individual variables and NSD.

## Materials and methods

### Study design and cohort

This retrospective study included 463 patients who underwent SSRO, comprising 873 rami, at the Department of Oral and Maxillofacial Surgery, Kyoto University Hospital.

### Inclusion and exclusion criteria

The inclusion criteria were SSRO underwent between January 1, 2014, and December 31, 2024. The exclusion criterion was incomplete follow-up data due to a lack of complete computerized information.

### Data collection

**Fig 1:**
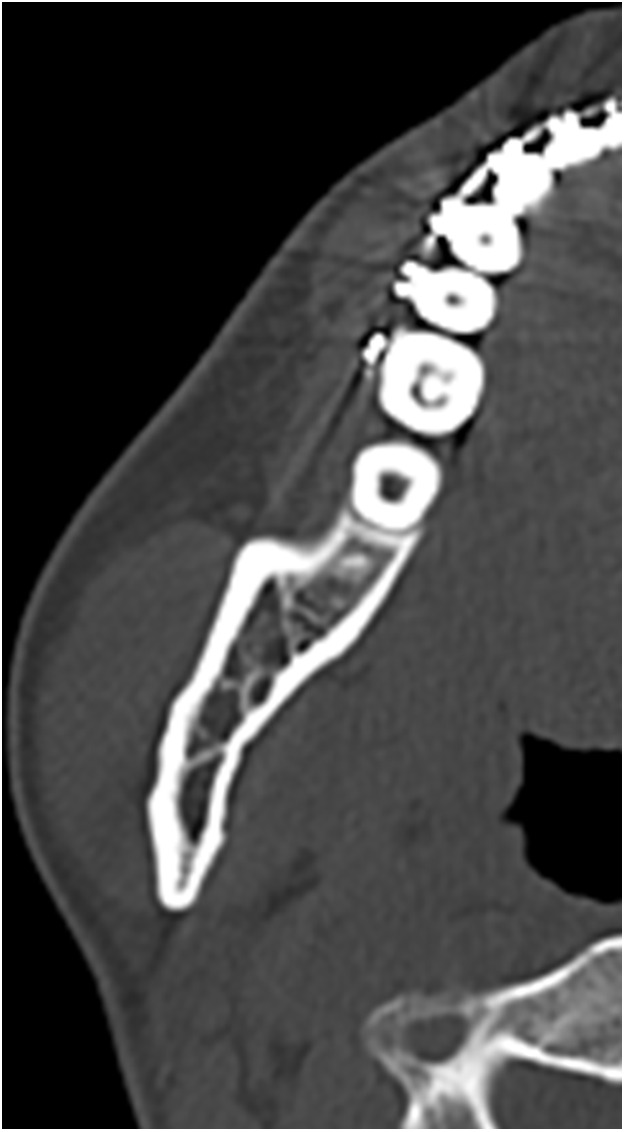
Axial computed tomography images showing no contact between the mandibular canal and the lateral cortical bone.

**Fig 2:**
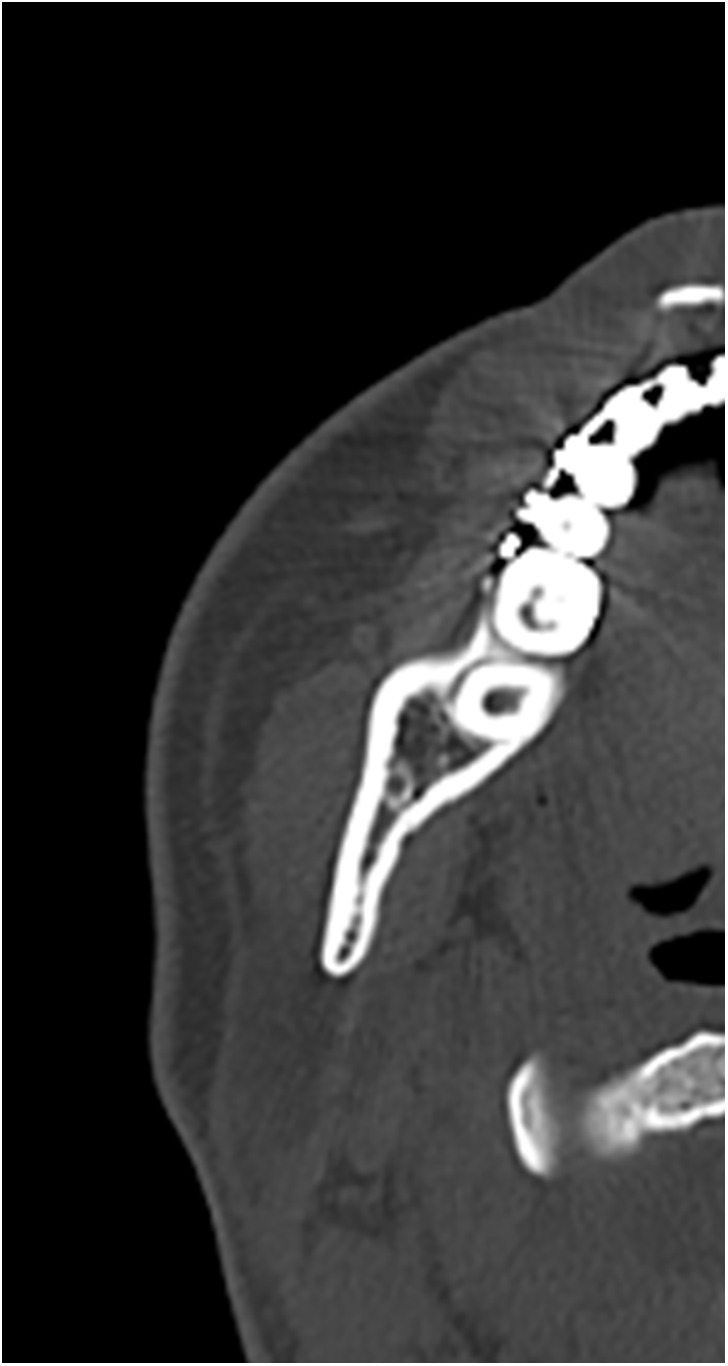
Axial computed tomography images showing contact between the mandibular canal and the lateral cortical bone.

**Fig 3:**
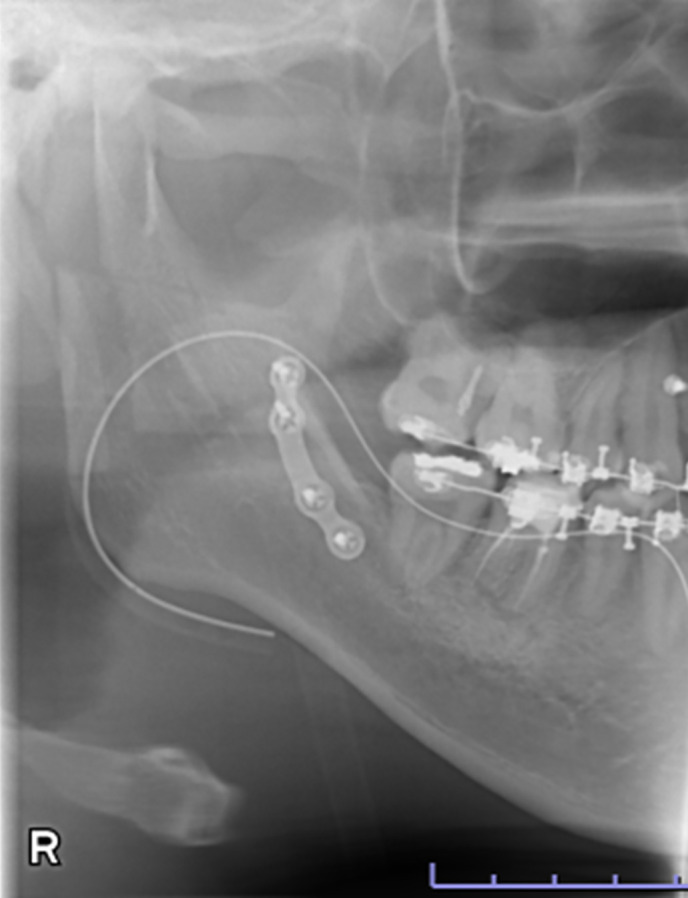
Panoramic radiograph showing the lateral osteotomy line in Trauner–Obwegeser method.

**Fig 4:**
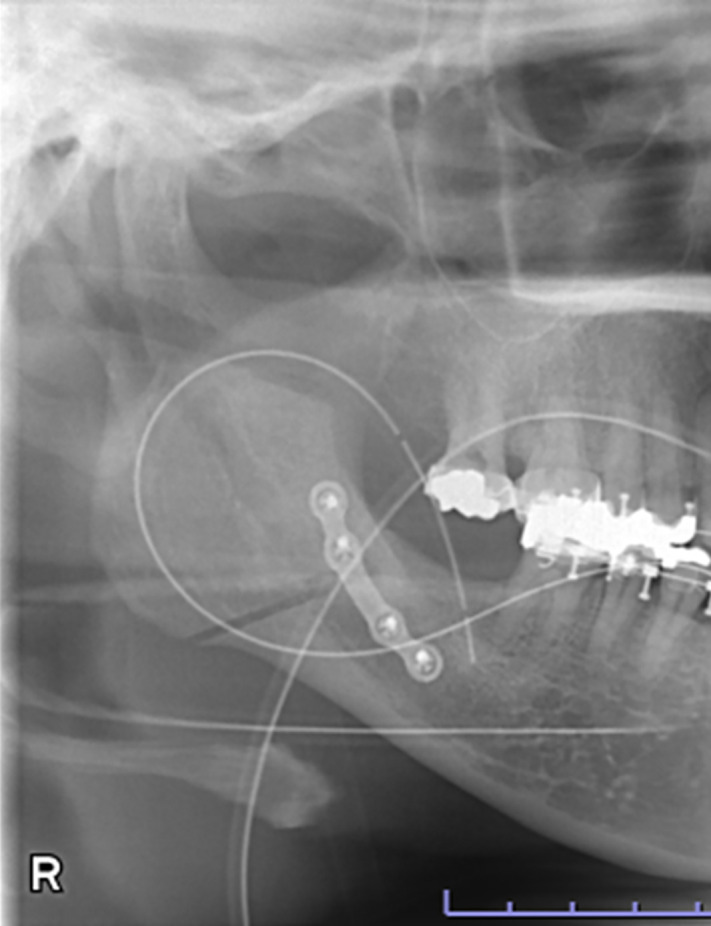
Panoramic radiograph showing the lateral osteotomy line in Obwegeser method.

**Fig 5:**
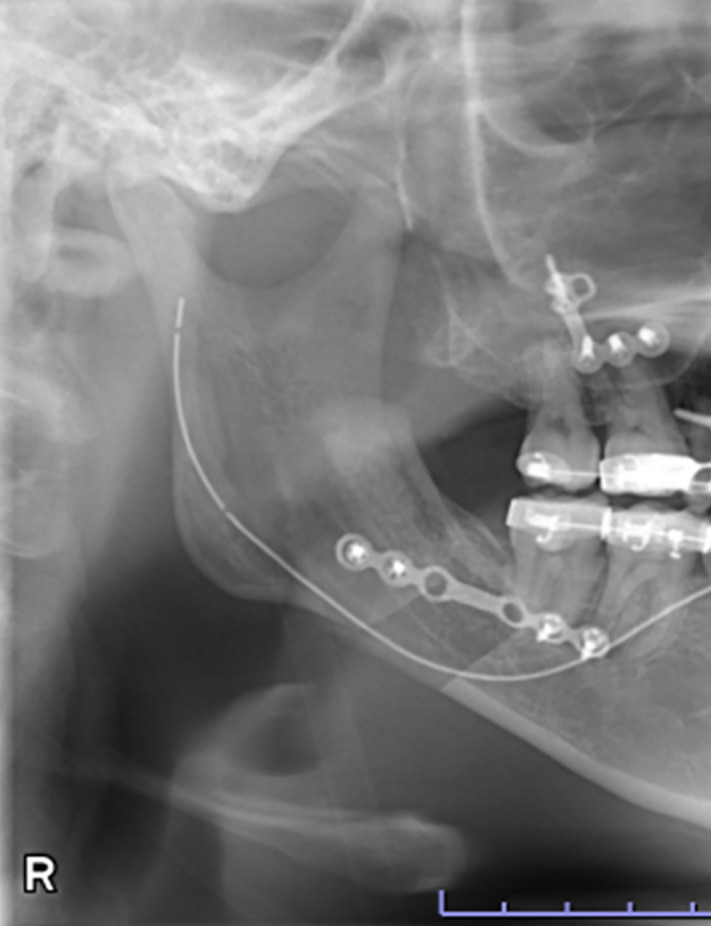
Panoramic radiograph showing the lateral osteotomy line in Obwegeser–Dal Pont method.

Data were retrospectively obtained from electronic medical records. Access to the database for data extraction was granted on May 11, 2025. The authors had no access to information that could identify individual participants during or after data collection. The characteristics of the study variables were categorized into patient-level and ramus-level variables, which were treated as explanatory variables. Patient-level variables included sex, age, body mass index (BMI) at the initial visit, and skeletal diagnosis, type of procedure, intraoperative blood loss, and operating time. Age was dichotomized at 30 years, consistent with prior studies defining young adulthood as 18–29 years and using 30 years as a boundary [14,15]. BMI was dichotomized at 25 kg/m² according to World Health Organization criteria (<25 vs. ≥ 25 kg/m²). Ramus-level variables included the side of the ramus undergoing SSRO, the presence or absence of contact between the mandibular canal (MC) and the lateral cortical bone (LCB), and the type of lateral osteotomy line (LOL). According to previous studies [16], based on preoperative computed tomography (CT) images, “MC–LCB contact” was defined as contact between the MC and the LCB at least once along the mandibular ramus (Figs 1 and 2). The LOL was assessed using postoperative panoramic radiographs and categorized into three types—Trauner–Obwegeser (TO), Obwegeser (Ob), and Obwegeser–Dal Pont (OD)—according to a previous study [17] (Figs 3–5). The outcome variable was NSD of the IAN. No patient exhibited NSD preoperatively. Postoperative NSD was assessed based on each patient’s most recent electronic medical record.

### Statistical analysis

Descriptive statistics for patient- and ramus-level variables, as well as the incidence of NSD of the IAN, are presented as numbers and percentages in tables. The annual number of ramus undergoing SSRO and those with NSD are summarized in a figure and analyzed using the Cochran–Armitage trend test. Univariable and multivariable logistic regression analyses were performed to examine associations between patient- and ramus-level variables and the outcome. Odds ratios (ORs), 95% confidence intervals (CIs), and *p*-value from both analyses are presented in tables. Variables included in the multivariable model were selected based on the clinical expertise of oral and maxillofacial surgeons and the results of the univariable analyses. All statistical analyses were conducted using JMP Student Edition version 19 (SAS Institute, Cary, NC, USA).

### Ethical approval

This study was conducted in accordance with the Declaration of Helsinki and was approved by the Institutional Review Board of Kyoto University Hospital (approval number: R4257). All patient records and information were anonymized and de-identified prior to analysis. The requirement for informed consent was waived because of the retrospective study design and the use of anonymized data. Information about the study was disclosed on the hospital’s website, and consent was obtained using an opt-out approach. Participants were given the opportunity to decline participation after reviewing the study information provided on the hospital’s website.

## Results

### Descriptive analysis of variables

This study included 463 patients who underwent SSRO, comprising 873 rami, over an 11-year period (2014–2024). Table 1 summarizes the characteristics of the study variables. Among the patient-level variables, 320 patients (69.1%) were female and 143 (30.9%) were male. The mean age was 27.1 years, and the mean BMI was 20.9 kg/m². The most common skeletal classification was class 3 (63.7%). 206 procedures (44.5%) were single-jaw surgeries and 257 (55.5%) were double-jaw surgeries. The median intraoperative blood loss was 270 mL, and the median operating time was 238 minutes. With respect to ramus-level variables, SSRO on the left side was 433 rami (49.6%), while the SSRO on the right side was 440 rami (50.4%). The mandibular ramus with MC–LCB contact was 138 rami (15.8%). The most common LOL was Ob method (93.0%).

**Table 1:** Characteristics of the study variables.

Patient-level (n = 463 cases)			
Sex			
	Female	320	(69.1)
	Male	143	(30.9)
Age (years)			
	Range		17–59
	Mean±SD		27.1 ± 8.1
	<30	336	(72.6)
	≥30	127	(27.4)
BMI (kg/m^2^)			
	Range		13.6-40.5
	Mean±SD		20.9 ± 2.9
	<25	427	(92.2)
	≥25	36	(7.8)
Skeletal Class			
	1	69	(14.9)
	2	99	(21.4)
	3	295	(63.7)
Procedure			
	Single-Jaw	206	(44.5)
	Double-Jaw	257	(55.5)
Blood Loss (ml)			
	Range		0-2590
	Median (Interquartile Range)	270	(125-500)
Operating Time (min)			
	Range		46-528
	Median (Interquartile Range)	238	(166-322)
**Ramus-level (n = 873 rami)**			
Side			
	Left	433	(49.6)
	Right	440	(50.4)
MC–LCB contact			
	(-)	735	(84.2)
	(+)	138	(15.8)
LOL			
	TO	30	(3.4)
	Ob	812	(93.0)
	OD	31	(3.6)

Data are presented as n (%) unless otherwise indicated.

SD = standard deviation; BMI = body mass index; SSRO = sagittal split ramus osteotomy; MC = mandibular canal; LCB = lateral cortical bone; LOL = lateral osteotomy line; TO=Trauner-Obwegeser; Ob = Obwegeser; OD = Obwegeser-Dal Pont.

Table 2 summarizes the incidence of NSD of the IAN: 5.6% (26/463) at the patient-level and 4.0% (35/873) at the ramus-level. Although the timing of postoperative assessments was not fully standardized, the median time to NSD assessment was 12 months (interquartile range, 8–14 months), with a mean of 13.3 ± 10.1 months.

**Table 2:** Incidence of NSD of the IAN.

		NSD of the IAN	
Patient-level	(n = 463 cases)	26	(5.6)
Ramus-level	(n = 873 rami)	35	(4.0)

Data are presented as n (%) unless otherwise indicated.

NSD=neurosensory disturbance; IAN=inferior alveolar nerve.

### Annual trends in neurosensory disturbance of the inferior alveolar nerve

**Fig 6:**
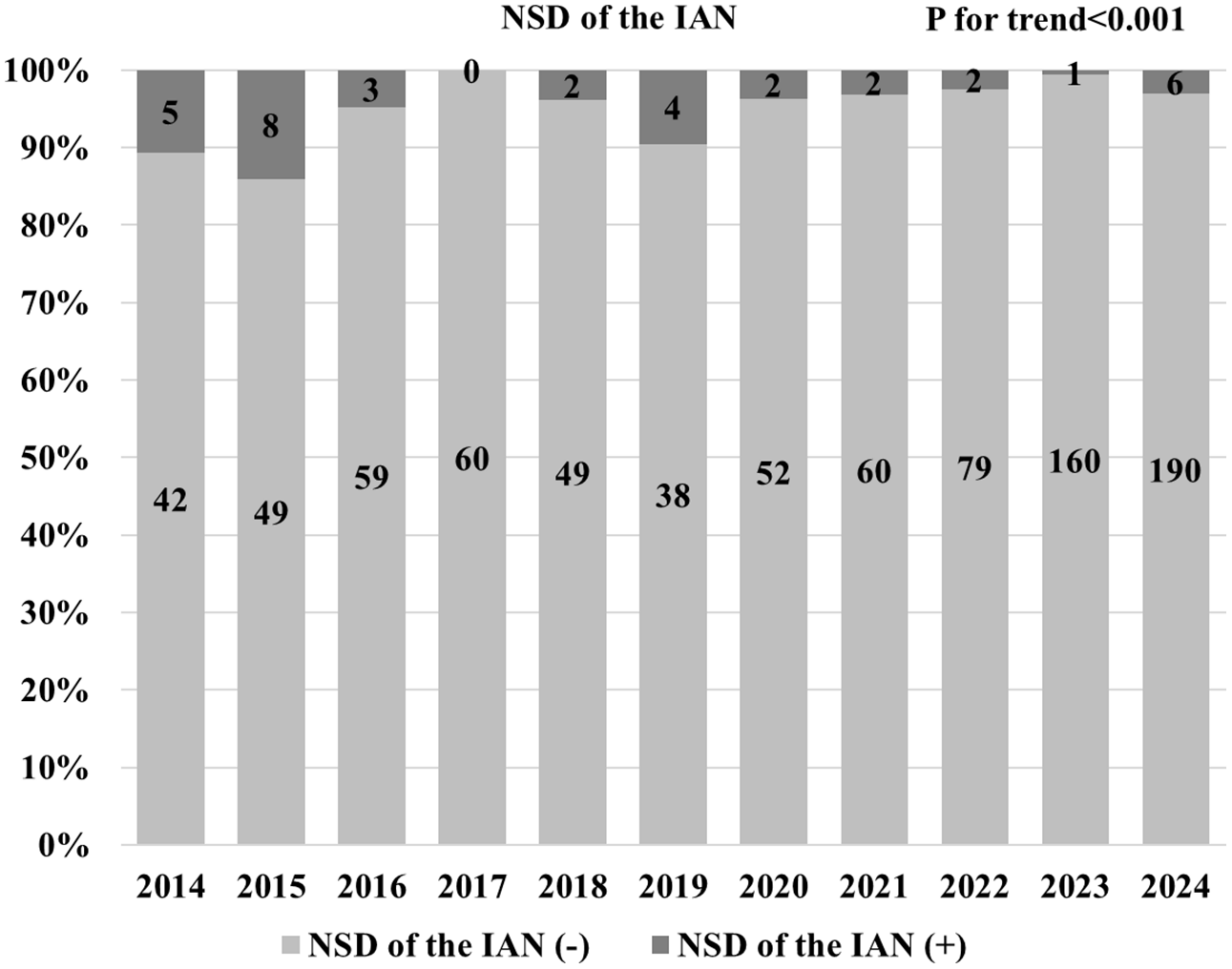
Annual number and proportion of ramus undergoing SSRO with NSD of the IAN. NSD = neurosensory disturbance; IAN = inferior alveolar nerve.

Fig 6 shows the annual number and proportion of ramus undergoing SSRO with NSD of the IAN. The proportion peaked in 2015 and subsequently showed a slight declining trend over time. A Cochran–Armitage trend test indicated a decreasing trend in the annual proportion of NSD following SSRO (*p* < 0.001).

### Logistic regression analyses for neurosensory disturbance of the inferior alveolar nerve

Table 3 summarizes the univariable and multivariable logistic regression analyses using patient-level variables performed to examine associations between each variable and NSD of the IAN. In the multivariable analysis, male sex tended to be associated with occurrence of NSD of the IAN (ref: female; OR, 2.05; 95% CI, 0.90–4.71; p = 0.09).

**Table 3:** Univariable and multivariable logistic regression analyses of patient-level predictors of NSD of the IAN.

Patient-level	Univariable			Multivariable		
	OR	(95%CI)	*p*-value	OR	(95%CI)	*p*-value
Sex (ref: Female)	2.36	(1.06, 5.28)	0.04	2.05	(0.90, 4.71)	0.09
Age (ref: < 30)	1.71	(0.73, 3.82)	0.21	1.80	(0.76, 4.07)	0.17
BMI (ref: < 25)	0.46	(0.03, 2.27)	0.40			
Skeletal Class 2 (ref: 1)	0.34	(0.05, 1.77)	0.20	0.42	(0.06, 2.27)	0.32
Skeletal Class 3 (ref: 1)	1.18	(0.43, 4.17)	0.76	1.15	(0.41, 4.09)	0.80
Skeletal Class 3 (ref: 2)	3.53	(1.00, 22.34)	0.05	2.75	(0.74, 17.85)	0.14
Procedure (ref: Single-Jaw)	0.93	(0.42, 2.09)	0.86			
Blood Loss (ref: < 270)	1.17	(0.53, 2.63)	0.69			
Operating Time (ref: < 238)	1.38	(0.62, 3.15)	0.43			

ref = reference; OR=odds ratio; CI = confidence interval; BMI = body mass index.

Table 4 summarizes the univariable and multivariable logistic regression analyses using ramus-level variables performed to examine associations between each variable and NSD of the IAN. In the multivariable analysis, MC–LCB contact was strongly associated with NSD of the IAN (OR, 4.96; 95% CI, 2.41–10.06; *p* < 0.001).

**Table 4:** Univariable and multivariable logistic regression analyses of ramus-level predictors of NSD of the IAN.

Ramus-level	Univariable			Multivariable		
	OR	(95%CI)	*p*-value	OR	(95%CI)	*p*-value
Side (ref: Left)	0.82	(0.41, 1.62)	0.57	0.77	(0.38, 1.53)	0.45
MC–LCB contact (ref: (-))	4.94	(2.45, 9.87)	<0.001	4.96	(2.41, 10.06)	<0.001
LOL Ob (ref: TO)	1.15	(0.23, 20.82)	0.89	1.87	(0.36, 34.53)	0.52
LOL OD (ref: TO)	3.11	(0.37, 64.88)	0.31	3.18	(0.37, 67.81)	0.31
LOL OD (ref: Ob)	2.70	(0.62, 8.17)	0.16	1.70	(0.38, 5.46)	0.45

ref = reference; OR=odds ratio; CI = confidence interval; BMI = body mass index; NSD = neurosensory disturbance; IAN = inferior alveolar nerve; MC = mandibular canal; lateral cortical bone = LCB; lateral osteotomy line = LOL; Trauner-Obwegeser=TO; Obwegeser = Ob; Obwegeser-Dal Pont = OD.

## Discussion

In this single-center retrospective study, we examined associations between patient- and ramus-level variables and NSD of the IAN following SSRO. The patient-level incidence of NSD was 5.8%, while ramus-revel incidence was 4.0%. A Cochran–Armitage trend test demonstrated a decreasing trend in the annual proportion of NSD cases (*p* < 0.001). Multivariable logistic regression analysis at ramus-level suggested that MC–LCB contact was strongly associated with NSD of the IAN (OR, 4.96; 95% CI, 2.41–10.06; p < 0.001).

Regarding the assessment of NSD of the IAN, both subjective methods, such as questionnaires, and objective methods, such as two-point discrimination tests, are used to evaluate sensory function and the presence of NSD [3,8]. Whether subjective or objective assessment should be considered the gold standard for NSD remains under debate [18]. However, Shibata et al. suggested that subjective assessment methods may be acceptable, as clinical decision-making regarding NSD should also incorporate patients’ subjective reports of sensory changes, and because the substantial discrepancy between subjective and objective assessments observed within the first 3 months postoperatively diminishes by 1 year after surgery [18–20]. Because patients’ subjective perception of numbness directly affects quality of life, the present study relied on electronic medical records primarily reflecting patient-reported symptoms at approximately 1 year after surgery.

A retrospective study reported that the incidence of long-lasting NSD after SSRO was 11.6% (10/86) at the ramus-level [5]. A systematic review reported that, at 1 year postoperatively, the frequency of IAN NSD after SSRO was 12.8% when assessed using objective methods and 23.8% when assessed using subjective methods at the ramus-level [19]. A multicenter prospective study reported that NSD of the IAN following SSRO occurred in 5.1% of cases at the ramus-level and in 8.9% at the patient-level [7]. Another retrospective study reported that, after a minimum follow-up period of 1 year, the incidence of NSD after SSRO at the ramus-level was 13.3% based on subjective assessment using questionnaires and 6.7% based on objective testing using cotton swabs and pin-prick testing [3]. In a retrospective study involving 263 patients (526 rami), NSD was observed in 27 rami (5.1%) at the ramus-level and in 26 patients (9.9%) at the patient-level at 1 year after SSRO [8]. Similarly, another retrospective study of 237 patients (474 sides) treated with SSRO reported NSD in 62 sides (13.1%) at the ramus-level and in 51 patients (21.5%) at the patient-level at 1 year postoperatively [18]. Taken together, these studies report an average incidence of NSD of 13.4% at the patient-level and 11.4% at the ramus-level. The incidence at the patient- and ramus- level observed in our cohort was lower than these values, at approximately 5%. A key strength of this study is its larger sample size compared with previous reports.

The incidence of NSD has decreased over time, likely reflecting advances in surgical techniques and instrumentation [6]. Medial dissection of the mandibular ramus during SSRO is technically demanding and requires adequate training, which may explain why less experienced surgeons demonstrate poorer IAN sensory outcomes than experienced surgeons [11]. Consistently, previous studies have reported a higher frequency of postoperative NSD of the lower lip and chin in patients treated by less experienced surgeons [21]. The use of piezoelectric cutting instruments may reduce the risk of intraoperative IAN injury, particularly when the nerve is located in close proximity to the outer cortical bone, owing to minimal damage to surrounding soft tissues [9]. A systematic review further suggested that piezoelectric surgery is associated with a lower incidence of macroscopic IAN injury compared with conventional techniques [9]. In contrast, conventional SSRO techniques may cause direct nerve injury during bone splitting with reciprocating saws or chisels [4]. In addition, a multicenter prospective study suggested that the use of a splitter and separator without a chisel may reduce the risk of IAN injury, as reflected by lower rates of postoperative hypoesthesia [7]. At our department, increasing specialist involvement over time and a shift from conventional instruments to ultrasonic bone-cutting devices and sagittal split separators may have contributed to the recent decline in NSD incidence by reducing trauma to surrounding tissues and the IAN.

With regard to the association between the MC and the LCB, one study reported that the MC contacted the external cortical bone in 10 of 40 rami (25%) [22]. Another study reported MC–LCB contact or fusion in 16 of 70 rami (22.9%), often extending from the mandibular foramen to the mandibular angle [16]. Several studies have identified a shorter distance between the MC and the buccal cortical bone as a risk factor for NSD [16,22–25]. In particular, the risk of NSD has been reported to increase significantly when this distance is ≤ 2 mm [23]. In our study, MC–LCB contact was observed in 15.8% of cases, which was relatively low and may reflect a previous preference for intraoral vertical ramus osteotomy over SSRO in patients with this anatomical feature at our department. Consistent with previous reports, our findings suggest that MC–LCB contact is a risk factor for NSD following SSRO.

Regarding the association between osteotomy patterns and NSD, a previous study reported that different types of lingual fracture in bilateral sagittal split osteotomy, categorized into short and long split groups, did not affect NSD incidence at 12 months postoperatively [26]. In another study, Hu et al. performed three-dimensional cone-beam CT evaluations of the mandible in 273 patients (546 sides) undergoing bilateral sagittal split osteotomy [27]. They classified six types of lingual split and three types of lateral bone cut end and found that neither pattern was associated with NSD incidence [27]. To the best of our knowledge, no studies have investigated the association between the type of lateral cortical osteotomy and NSD in SSRO, as opposed to the type of medial osteotomy. At our department, the Ob method was used in most SSRO cases, and strong association between lateral osteotomy type and NSD was not observed. Regardless of the osteotomy line, careful and appropriate osteotomy in the second molar region is essential to avoid injury to the IAN, as the buccal bone thickness had its greater dimension buccal to the IAN in this region [28].

The main limitation of this study is its retrospective design. The analysis relied on the accuracy and completeness of data available in the electronic medical records, which may have introduced a risk of missing data because of gaps in information or incomplete documentation. In addition, reflecting a limitation inherent to retrospective studies relying on electronic medical records, the timing of assessments for NSD was not fully standardized. Finaly, this study was conducted at a single institution, which may limit the generalizability of the findings.

## Conclusion

This study evaluated a large cohort of patients undergoing SSRO and investigated the incidence and patient- and ramus-level risk factors for NSD of the IAN. The overall incidence of NSD was approximately 5.0% and showed a declining annual trend over time, possibly reflecting increasing surgical experience and improved instrumentation. MC–LCB contact on preoperative CT was associated with an approximately fivefold higher incidence of NSD, highlighting the importance of careful and gentle osteotomy in SSRO or consideration of alternative surgical procedures. Further studies incorporating additional factors are warranted to establish effective preventive strategies and improve patient outcomes.
